# Versatile networks of simulated spiking neurons displaying winner-take-all behavior

**DOI:** 10.3389/fncom.2013.00016

**Published:** 2013-03-19

**Authors:** Yanqing Chen, Jeffrey L. McKinstry, Gerald M. Edelman

**Affiliations:** ^1^The Neurosciences InstituteSan Diego, CA, USA; ^2^Mathematical, Information, and Computer Sciences, Point Loma Nazarene UniversitySan Diego, CA, USA

**Keywords:** brain-based computational model, spiking neuronal networks, winner-take-all, motor control and learning/plasticity, spike-timing-dependent plasticity, sensorimotor control, large-scale spiking neural networks, neurorobotics

## Abstract

We describe simulations of large-scale networks of excitatory and inhibitory spiking neurons that can generate dynamically stable winner-take-all (WTA) behavior. The network connectivity is a variant of center-surround architecture that we call center-annular-surround (CAS). In this architecture each neuron is excited by nearby neighbors and inhibited by more distant neighbors in an annular-surround region. The neural units of these networks simulate conductance-based spiking neurons that interact via mechanisms susceptible to both short-term synaptic plasticity and STDP. We show that such CAS networks display robust WTA behavior unlike the center-surround networks and other control architectures that we have studied. We find that a large-scale network of spiking neurons with separate populations of excitatory and inhibitory neurons can give rise to smooth maps of sensory input. In addition, we show that a humanoid brain-based-device (BBD) under the control of a spiking WTA neural network can learn to reach to target positions in its visual field, thus demonstrating the acquisition of sensorimotor coordination.

## Introduction

Analyses in computational neurobiology have successfully used mean-firing-rate neuronal models to simulate the spatiotemporal patterns of neural activity that arise in interconnected networks of excitatory and inhibitory neurons, such as those in the vertebrate cortex (Von der Malsburg, 1973; Obermayer et al., 1990; Dayan and Abbott, 2001). Certain aspects of these systems may, however, require the modeling of the dynamic properties of large populations of individual neurons, each calculated with millisecond precision. Simulations of such systems are challenged with issues such as non-linearity, instability, and resistance to scaling. Here we address these issues by simulating networks of spiking neurons that are capable of sensory map formation and sensorimotor interactions.

It has been proposed that local microcircuits of the cerebral cortex can function as winner-take-all (WTA) networks (Douglas and Martin, 2004). In such systems, an individual pattern of input can evoke network responses that suppress possible alternative responses. In addition, the population response to any sensory stimulus is sparse. This proposal is attractive for several reasons. On theoretical grounds, WTA networks have demonstrated utility in models of pattern recognition (Von der Malsburg, 1973), map formation (Obermayer et al., 1990), selective attention (Itti et al., 1998), and working memory (Wilson and Cowan, 1973). The proposal is also supported by cortical anatomy. A characteristic structural feature of WTA networks is long range inhibition among cellular components coupled to short range excitation. Anatomical evidence exists for such an architecture in animal nervous systems (Goldman-Rakic, 1995; Kisvárday et al., 2000; Holmgren et al., 2003; Fino and Yuste, 2011; Perin et al., 2011). Indirect physiological evidence (Derdikman et al., 2003; Haider et al., 2010) has also been obtained for local excitation and surround inhibition in the cerebral cortex of mammals.

Rate-based WTA networks with center-surround architecture have been extensively explored (Dayan and Abbott, 2001). Although these networks have been shown to possess useful properties, they lack the temporal precision and biological realism of networks of spiking neurons. In some prior studies of spiking models capable of WTA behavior the neuronal network structure has been highly simplified. Networks are simulated as a one-dimensional chain or ring (Laing and Chow, 2001; Shriki et al., 2003). The inhibitory population may be reduced to one unit (Oster et al., 2009; Rutishauser et al., 2011), or the inhibitory population was removed altogether and modeled as direct inhibitory connections among excitatory neurons (Laing and Chow, 2001; Choe and Miikkulainen, 2004). One large-scale spiking model did produce smooth maps of orientation columns, but this model also combined excitatory and inhibitory neurons into a single population, and did not incorporate spike-timing-dependent plasticity (STDP) (Choe and Miikkulainen, 2004). If the complex circuits of the cortex function as WTA networks, biologically realistic spiking models must exhibit robust WTA network dynamics that can explain behavior at the systems level.

**Figure 1:**
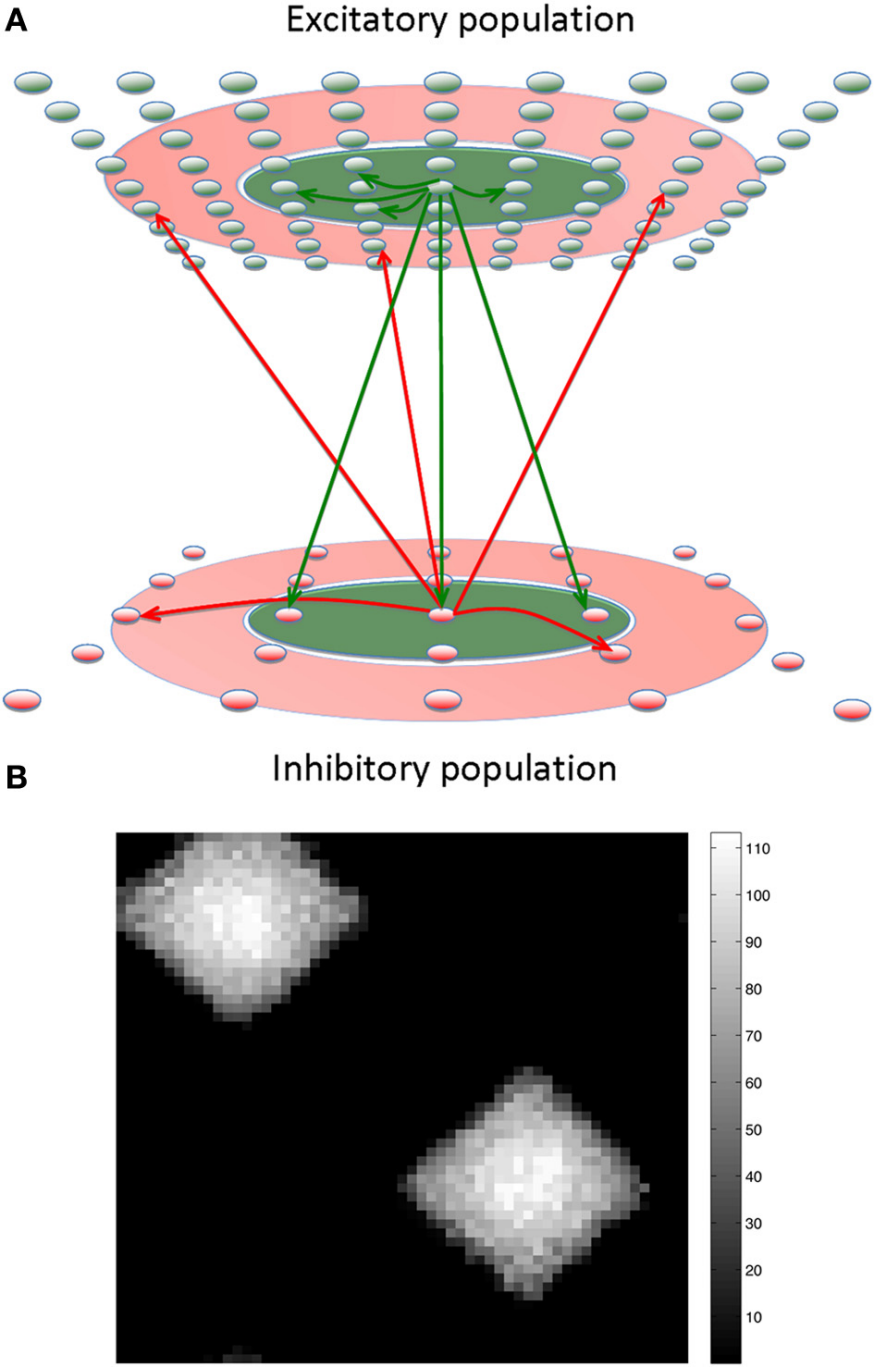
**The center-annular-surround (CAS) spiking network architecture leads to winner-take-all (WTA) dynamics. (A)** The CAS network architecture consists of interconnected spiking neurons, excitatory (green ovals), and inhibitory (red ovals). Each population is arranged in a two-dimensional grid. Connections from representative cells are illustrated. Axons from excitatory neurons (green arrows) project to neurons within green areas. Axons from inhibitory neurons (red arrows) project to neurons in the transparent red annular areas. The sensory input projecting non-topographically to both the excitatory and inhibitory “cortical” populations is not shown. **(B)** The CAS connectivity leads to WTA dynamics: small areas of high activity are surrounded by large regions with little activity. The firing rates of excitatory neurons in the network are shown as pixels with brightness proportional to firing-rate indicated by the scale bar to the right (in Hz). The number and size of the winning regions are functions of a variety of network parameters.

In the present study we describe a general and robust computer simulation of the activity within neural networks containing thousands of excitatory and inhibitory spiking neurons in a variant of center-surround architecture that we call center-annular-surround (CAS). In this architecture each neuron is excited by nearby neighbors and inhibited by more distant neighbors in an annular-surround region (Figure 1A). The neural units of these networks simulate conductance-based spiking neurons that interact via mechanisms susceptible to both short-term synaptic plasticity and STDP. We show that such CAS networks display robust WTA behavior unlike the center-surround networks we have studied. We demonstrate for the first time that a large-scale network of spiking neurons with separate populations of excitatory and inhibitory neurons can give rise to smooth maps of sensory input (Obermayer et al., 1990). We also show that, a brain-based-device (BBD) under the control of a system of such networks learns to reach to visual targets.

## Materials and methods

### Spiking neuronal networks

Each modeled network (Figure 1A) is comprised of three interconnected populations of spiking neuronal units (Izhikevich, 2010) distributed over two-dimensional square grids. Each population is composed of units simulating one of three functional classes of spiking neurons: input (“thalamic”), excitatory, and inhibitory. The parameters of simulated neurons in each class are tuned so that the voltage waveform mimics its biological counterpart (Izhikevich, 2003). The synapses display STDP and short-term plasticity dynamics as previously described in detail (Izhikevich and Edelman, 2008). The neuron model equations, short-term synaptic plasticity equations, and STDP equations are presented below.

### Neuronal dynamics

Spiking dynamics of each neuron were simulated using the phenomenological model proposed by Izhikevich (2003). The model has only two equations and four dimensionless parameters that could be explicitly determined from neuronal resting potential, input resistance, rheobase current, and other measurable characteristics. We present the model in a dimensional form so that the membrane potential is in millivolts, the current is in picoamperes and the time is in milliseconds:

(1) $$C\dot{v}=k(v-v_{r})(v-v_{t})-u-I_{\text{syn}}$$

(2) $$\dot{u}=a\left\{b(v-v_{r})-u\right\}$$

where *C* is the membrane capacitance, *v* is the membrane potential (in mV), *v*_*r*_ is the resting potential, *v*_*t*_ is the instantaneous threshold potential, *u* is the recovery variable (the difference of all inward and outward voltage-gated currents), *I*_syn_ is the synaptic current (in pA) defined below, a and b are parameters. When the membrane potential reaches the peak of the spike, i.e., *v* > *v*_peak_, the model fires a spike, and all variables are reset according to *v* ← *c* and *u* ← *u* + *d*, where *c* and *d* are parameters. Table A1 lists each of the neuron model parameters used in all experiments. At the start of all simulations, *v* was set to −60 for all neurons, whereas *u* was set to a different random value for each neuron drawn uniformly from the range 0–100.

### Short-term synaptic plasticity

The strength of synapses varied as a function of the presynaptic neuron's firing history. We assume that the synaptic conductance (strength) of each synapse can be scaled down (depression) or up (facilitation) on a short time scale (hundreds of milliseconds) by a scalar factor *x*. This scalar factor, different for each presynaptic cell, is modeled by the following one-dimensional equation

(3) $$\dot{x}=(1-x)/{\text{τ}}_{x},\;x\leftarrow px\text{ when presynaptic neuron fires}.$$

*x* tends to recover to the equilibrium value *x* = 1 with the time constant τ_*x*_, and it is reset by each spike of the presynaptic cell to the new value *px*. Any value *p* < 1 decreases *x* and results in short-term synaptic depression, whereas *p* > 1 results in short-term synaptic facilitation. The parameters, τ_*x*_ and *p*, for each combination of presynaptic and postsynaptic neuron types were as follows: exc. → exc.: 150, 0.8; exc. → inh.: 150, 0.8; inh. → exc.: 150, 0.8; inh. → inh.: 150, 0.8; thalamic → exc: 150, 0.7; thalamic → inh.: 200, 0.5.

### Synaptic kinetics

The total synaptic current to each neuron is simulated as

(4) $$I_{\text{syn}}=g_{\text{AMPA}}(v-0)+g_{\text{NMDA}}\frac{{\left[(v+80)/60\right]}^{2}}{1+{\left[(v+80)/60\right]}^{2}}(v-0)+\;g_{{\text{GABA}}_{\text{A}}}(v+70)+g_{{\text{GABA}}_{\text{B}}}(v+90)+g_{\text{SH}}(v+90)$$

where *v* is the postsynaptic membrane potential, and the subscript indicates the receptor type. Each conductance *g* (here we omit the subscript for the sake of clarity) has first-order linear kinetics *g*′ = −*g*/τ with τ = 5, 150, 6, 150, and 5000 ms for each of the simulated AMPA, NMDA, GABA_A_, GABA_B_, and slow hyperpolarizing (SH) receptors, respectively. The SH “receptors” were an *ad hoc* method for adding SH currents in order to bias cells to remain off for longer periods of time; this improved pattern separation, and was used only in the BBD experiments.

Each firing of an excitatory neuron increases *g*_AMPA_ by *xc*, where *c* is the synaptic conductance (synaptic weight) in nanoSiemens and *x* is the short-term depression/potentiation scaling factor as above; *g*_NMDA_ was increased by *nmda_gain xc*, where *nmda_gain* is the ratio of NMDA to AMPA conductances and is found experimentally to be less than one (Myme et al., 2003). Similarly *gabab_gain* and *gabash_gain* are used to adjust the contribution of *g*_GABAB_ and *g*_SH_, respectively, relative to *g*_GABAA_. The gain factor for *g*_*SH*_ was set to zero for all simulations except for the BBD experiments in which case the gain factor was set to 0.2 for the first 45 simulation seconds and was set to 0.0 for the remainder of the simulation.

### STDP

The change in conductance (weight) of each synapse in the model is simulated according to STDP: the synapse is potentiated or depressed depending on the order of firing of the presynaptic and postsynaptic neurons (Bi and Poo, 1998). We use the following equations to update each plastic synapse, *s*, in the network:

(5) $$\dot{c}=-c/\tau_{c}+\alpha \text{STDP}(t)\delta (t-t_{\text{pre}/\text{post}})$$

(6) $$\dot{s}=c$$

where δ (*t*) is the Dirac delta function that step-increases the variable *c*. Firings of pre- and postsynaptic neurons, occurring at times *t*_pre_, *t*_post_, respectively, change *c* by the amount αSTDP(*t*) where α is the learning rate for the synapse, *t* = *t*_post_ − *t*_pre_ is the interspike interval, and

(7) $$\text{STDP}(t)=\left\{\begin{array}{cc} A^{+}\exp{\left(-1/\tau^{+}\right)}^{t}, & t>0\\ A^{-}\exp{\left(-1/\tau^{-}\right)}^{\left|t\right|}, & t\leq 0 \end{array}\right\}.$$

where *A*^+^ = 0.005, *A*^−^ = 0.001, τ^+^ = τ^−^ = 20 ms. The variable *c* decays to zero exponentially with the time constant τ_*c*_ = 1 s, and s is updated once every 50 ms for computational efficiency. Note that for simplicity, each synapse was modeled with a single weight, *s*; therefore the STDP rule changed both AMPA and NMDA components of the synapse proportionally.

### Synaptic scaling

Synaptic scaling was performed for each neuron in order to maintain the total of all synaptic strengths on a given connection pathway, *s*_total_, at a constant value. This scaling was performed for every neuron every 50 ms during the simulation. In addition, each synapse was prevented from exceeding *s*_max_ or going below zero, regardless of learning rules and normalization.

### Anatomy

The Input network is composed of 484 simulated “thalamic” neurons that provide excitatory input to “cortical” excitatory and inhibitory neurons. “Thalamic” neurons project to both “cortical” populations with uniform random connectivity. Current levels to these “thalamic” cells were adjusted to evoke distinct patterns of activity in the input area with a maximum firing rate of approximately 100 Hz for either abstract patterns or video camera input.

The cortical network contained 3481 excitatory cells and 900 inhibitory cells. All connections made from cortical excitatory neurons to other neurons followed local-type connectivity. In this connectivity, a two-dimensional Gaussian probability distribution, centered on each cell, determined the probability of forming an input synapse to surrounding neurons. This probability density function was scaled to generate, on average, a pre-specified number of excitatory synapses onto each cell (see Appendix for details). The initial synaptic strength between connected neurons also varied as a Gaussian function of the distance between them. The total of all synaptic efficacies for each simulated neuron was scaled to sum to a constant value unique to each neuron type.

In contrast, inhibitory neurons in the system exhibited CAS connectivity. For CAS connectivity, each neuron received synaptic input only from neurons located in a surrounding area specified by a minimum (*r*_min_) and maximum (*r*_max_) radial distance from the postsynaptic cell. The probability of forming a connection with a neuron in the annulus was a function of the distance separating the cells. The function used was a Gaussian with standard deviation σ, centered at (*r*_min_ + *r*_max_)/2. This probability distribution function was scaled to create a prespecified number of inhibitory synapses onto each neuron. The synaptic strengths for the surround-type connection were also initialized using the same function, with the same parameters. However, the synaptic strengths of this type were scaled to make their sum equal to a constant value under experimental control.

We found that this CAS connectivity arrangement confers WTA properties to the networks. Each distinctive pattern of neural activity in the “thalamic” network evoked enhanced neural activity in only a few localized patches in the “cortical” area due to competitive interactions between local neural populations (Figure 1B). Local patches of interconnected neurons that on average respond better than surrounding cells “win” a dynamic competition and remain active. In contrast, neurons in the surround are suppressed by inhibition and do not fire. A detailed description of the network along with all the parameter settings used in the experiments can be found in the Appendix, and connectivity parameters can be found in Tables A2–A4.

### Winner-take-all measure

We use the following measure of population sparseness (*S*) to characterize WTA dynamics in the excitatory population:

$$S=\frac{1-{\left(\sum_{j=1}^{N}\frac{r_{j}}{N}\right)}^{2}/\left(\sum_{j=1}^{N}{\frac{r_{j}}{N}}^{2}\right)}{1-\frac{1}{N}}$$

where *r*_*j*_ is the number of spikes emitted by neuron *j* during the measurement interval, (one second in this paper) and *N* was the number of neurons in the population (Willmore and Tolhurst, 2001).

### Brain-based-device (BBD)

To demonstrate that a simulated network can control real-world behavior, we designed and constructed a humanoid BBD. The device is 50 cm high and uses a black and white wireless webcam for vision. Each arm of the BBD contains eight Dynamixel servomotors (Robotis, Irvine, CA, USA). In the specific experiments described here only the two shoulder joints function; all other joints remain stationary with the arm extended. Shoulder joint angles provided by the motors determine the posture of the arms. A miniature PC (VIA Technologies, Fremont, USA) mounted on the back of the BBD maintains wireless communication between the device and the neuronal networks simulated on a Mac-Pro *(Apple, Inc. Cupertino, CA)*.

A simulated motor neural network constructed and incorporated into the BBD controlled its behavior. This network was similar to the sensory network, but was composed of only 1600 excitatory and 400 inhibitory spiking neurons. Different patterns of activity in the motor area neurons specified distinct equilibrium postures of the left arm. Since the camera of the BBD was aimed at the left robotic hand, each of these postures presented a distinct pattern of visual input to the system. The motor region received non-topographic connections from the output of the sensory network. By adjusting parameters of feed-forward connections to the motor area from the cortical area receiving camera input, this system came to associate the visual input evoked by different postures to the motoric output pattern that would generate and maintain those postures.

### Position error calculation

We measure the position error of a given joint as follows. For every arm posture measured during testing, we find the closest posture found during the training period. We then measure the angular difference of the joint between these two postures. We report the median and the maximum joint position error across all joints, reaching trials, and subjects.

## Results

### Spiking activity in a WTA network

We first characterized spiking activity in the network as a function of the parameters of network connectivity (Figure 1). All analyses were carried out under the assumption of CAS connectivity described above, and examined the effects of different patterns of relative synaptic strengths on the various pathways in the network.

In these analyses, each simulated network received identical random input to “thalamic” cells and started with identical random neural states, but had different values of total excitatory-to-inhibitory and inhibitory-to-excitatory synaptic strengths. The total weight of inhibitory-to-inhibitory synapses was kept equal to 2.4 times the total weight of inhibitory-to-excitatory synapses to limit the parameter space. The strengths of excitatory-to-excitatory synapses were kept constant in all simulations. Connection strengths were not modulated by STDP but were subject to the short-term synaptic plasticity inherent in modeled neurons (Izhikevich and Edelman, 2008). Exact values of all parameters are given in Table A2. All spikes that the networks emitted between 2 and 3 s after the onset of thalamic input were recorded, at which time most simulations had reached steady state.

**Figure 2:**
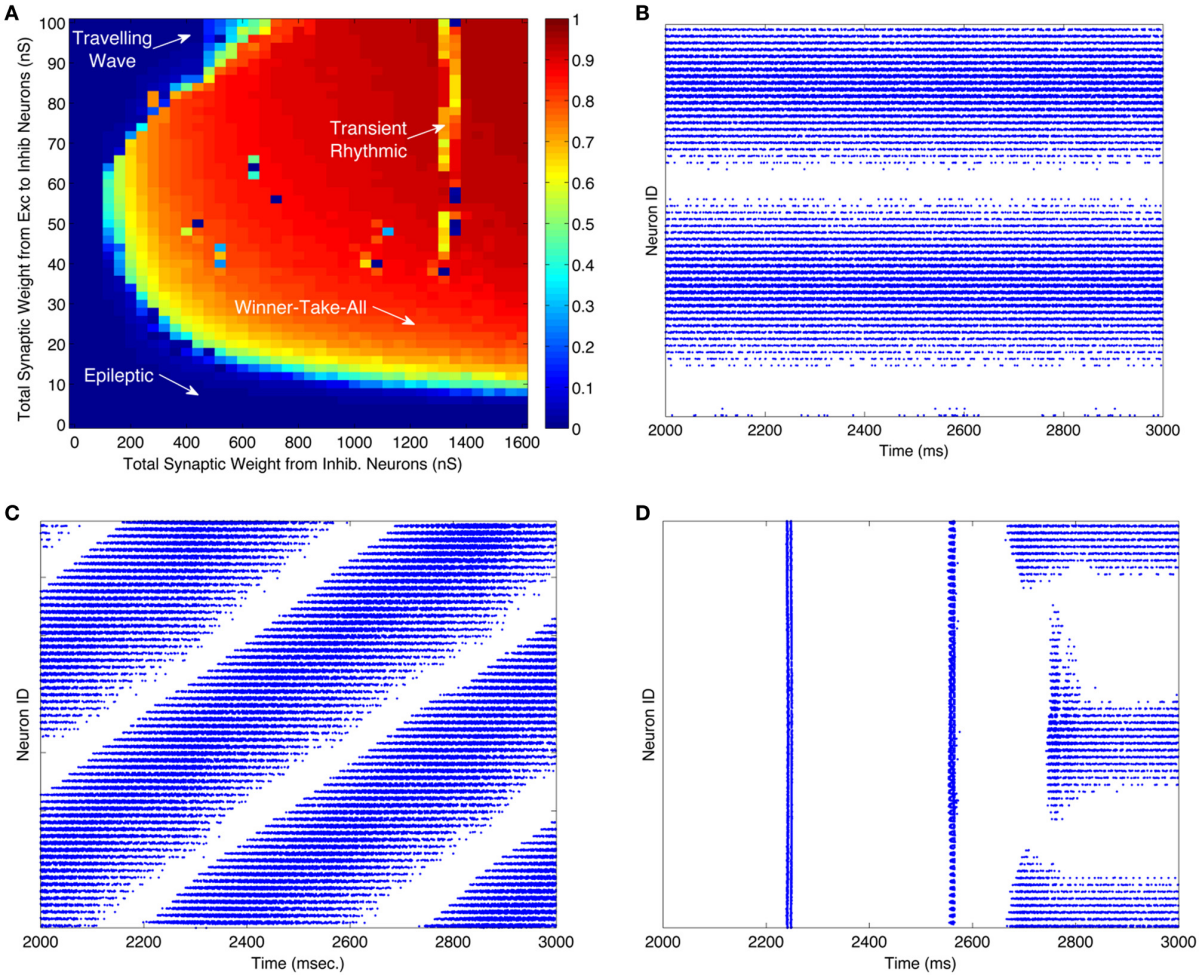
**WTA dynamics can occur in large regions of the parameter space of CAS networks. (A)** A measure of winner-take-all behavior in a network is plotted as a function of synaptic weights coupling the excitatory and inhibitory neural populations. The measure we use is the highest firing rate of any neuron in the network, subject to a sparseness constraint that at least half of the neurons in the network are firing at less than 2 Hz; otherwise the measure is defined to be zero. The total synaptic conductance in nano-siemens (nS) (Izhikevich and Edelman, 2008) in each individual inhibitory neuron from excitatory neurons is on the y-axis, and total inhibitory conductance received by each neuron, excitatory or inhibitory, is on the x-axis. The orange and red areas indicate regions of the parameter space in which the network exhibits WTA behavior. The lower left region of the parameter space, labeled “Epileptic,” defines networks exhibiting epileptic dynamics in which all neurons fire indiscriminately to the stimulus. **(B–D)** Are raster plots which show all spikes (blue dots) during the third second of the simulation for each excitatory neuron in the network. **(B)** All spikes of a network in a WTA state at parameters labeled “Winner-Take-All” in (panel **A**). During this state some excitatory neurons (horizontal band of blue dots) fire persistently in response to a constant stimulus while others are silent. **(C)** At certain values of parameters, labeled “Traveling Wave” in (panel **A**), region of the parameter space, the network exhibits moving patches of activity instead of the stable patches shown in **(B)**; this results in diagonal bands in the one-dimensional raster plot. **(D)** Occasionally the network requires more than two simulated seconds for a winner to emerge. These “Transient Rhythmic” states result in all neurons firing synchronously and rhythmically for some time before a winning group emerges. See Figures A1–A3 for close up plots from (panels **B–D**). A raster plot corresponding to the epileptic activity state is not shown.

Figure 2A illustrates the dynamic behavior of these networks for 2000 different combinations of excitatory-to-inhibitory and inhibitory-to-excitatory synaptic strengths. The color of each pixel in Figure 2A is determined by a measure of the WTA behavior of the network dynamics in the same 1 s time period. Since WTA behavior entails sparse activity, we use a standard measure of population activity sparseness to characterize WTA behavior (see “Materials and Methods”). The measure will be close to one for networks in which only a small subset of neurons respond to the “thalamic” input with elevated firing rates. Parameters modeled in each raster plot in Figures 2B–D are indicated by a corresponding labeled arrow in Figure 2A.

When both excitatory and inhibitory connection weights were relatively high, local patches of excitatory neurons had a high maximal firing rate, as shown in the corresponding spike raster plot (Figure 2B). However, only a localized subset, (25% of this neuronal population), maintained high firing rates; most neurons were silent. This outcome, in which a subset of neurons fires persistently at a high frequency and suppresses the activity of other neurons, defines a WTA network state. The majority of the parameter space explored corresponds to the WTA state as indicated by the predominance of warm colors in Figure 2A.

**Figure A1:**
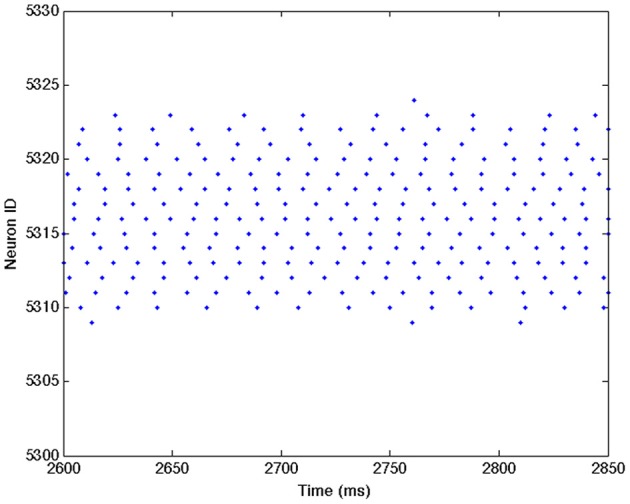
**Close up of Figure 2B showing individual spike trains for a small subset of excitatory neurons**. See Figure 2B caption for a complete description.

**Figure A2:**
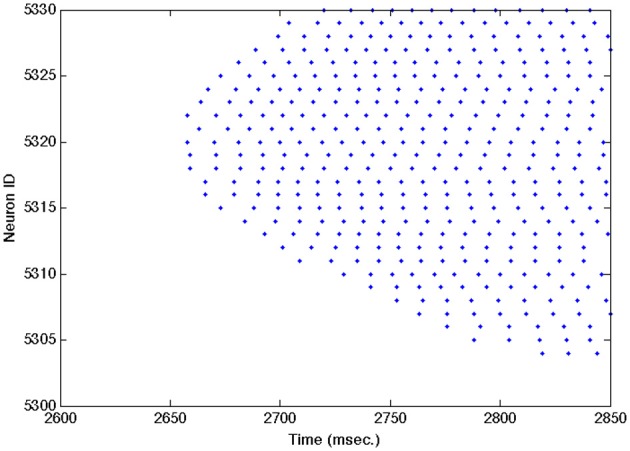
**Close up of Figure 2C showing individual spike trains for a small subset of excitatory neurons**. See Figure 2C caption for a complete description.

**Figure A3:**
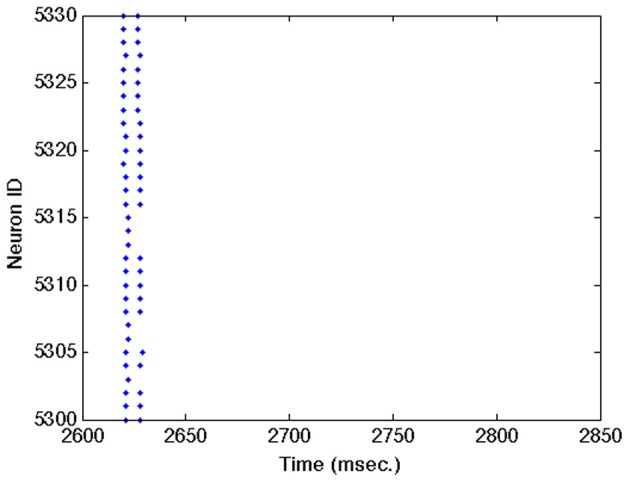
**Close up of Figure 2D showing individual spike trains for a small subset of excitatory neurons**. See Figure 2D caption for a complete description.

The spike raster plot in Figure 2C shows activity within a network in a traveling wave state. The firing of both excitatory and inhibitory neurons moves as a localized “patch” through the network rather than remaining stationary in a WTA state. Figure 2D shows a network that remained in an initial rhythmic, periodic state for a prolonged period after stimulus onset, but entered a WTA state toward the end of the third second of stimulus presentation. Single excitatory neurons maintained a state of high-frequency spiking activity only when connection strengths were within the WTA region delineated in Figure 2A. Figures A1–A3 show close up plots from portions of Figures 2B–D.

For comparison, we also simulated the spiking behavior of networks of excitatory and inhibitory cells linked together in three different, non-CAS architectures. The three alternative network architectures analyzed were: (1) standard center-surround architecture in which connectivity among all neurons was determined by a two-dimensional Gaussian probability distribution centered on each cell, inhibition having a larger σ than excitation; (2) an inverse connectivity in which the excitatory connections project to an annular-surround and the inhibitory neurons connect locally, and (3) uniform random connectivity among all neuron types (excitatory-to-inhibitory, inhibitory-to-excitatory, excitatory-to-excitatory, and inhibitory-to-inhibitory) (see Appendix for details of the parameters used). In the same parameter space analyzed in Figure 2A, none of these connection types supported WTA behavior, characterized by stable patch activity. The maximum population sparseness measure for the three alternative network architectures listed above were 0.16, 0.54, and 0.21, respectively, whereas for the CAS network, the majority of the parameter space yielded population sparseness measures close to 1 (Figure 2A). The most common firing patterns evoked in these neural networks were quasi-rhythmic firings of excitatory neurons in the 10–20 Hz range punctuated with short bursts of localized activity in inhibitory neurons. Among the different connectivities we analyzed, only the CAS motif gave rise to localized persistent activity that defines a WTA state.

### Using CAS architecture to develop maps of orientation selectivity

Smooth maps, in which nearby neurons have similar response properties, are ubiquitous in sensory and motor regions of the cerebral cortex (Obermayer et al., 1990; Kaschube et al., 2010). For example, in the primary visual area of many animals, smooth retinotopic maps coexist with smooth maps of stimulus orientation. Computational neural models have successfully generated such smooth maps (Choe and Miikkulainen, 2004), but not, so far, with detailed networks of excitatory and inhibitory spiking neurons. It is therefore of interest to investigate whether such simulated networks of interconnected excitatory and inhibitory spiking neurons might produce such maps. We found that by slowly increasing inhibition in the model over time as experimentally observed (Ben-Ari et al., 2012), the CAS network described above develops smooth orientation maps when trained with oriented visual input.

The “thalamic input” to the “cortical” cells were given a rough initial topographic bias (Choe and Miikkulainen, 2004) by limiting the maximum distance over which “thalamic” inputs traveled to synapse on “cortical” cells. This simulation allows a maximum radius of 0.65 mm in a simulated 2 mm by 2 mm cortical region. Training stimuli consisted of 4000 images of computer-generated elongated Gaussian shapes distributed throughout the visual field at random locations and orientations as in Choe and Miikkulainen (2004). STDP was used to train a network of 60 by 60 excitatory and 30 by 30 inhibitory neurons for 40,000 simulated seconds. Each of the 4000 stimuli was presented to the network 20 times, and each presentation lasted 500 ms.

To assure smoothness in the resulting maps, more abstract models of orientation map formation have generally made use of an annealing process (Obermayer et al., 1990). This annealing process takes the form of a slow decrease of the size of the subpopulation of neurons active during the presentation of a stimulus (Kohonen, 1984). We sought a biological mechanism to accomplish this slow decrease in the active population size. Recent experimental evidence suggests that early during development, GABAergic conductances are excitatory rather than inhibitory (Ben-Ari et al., 2012). We hypothesized that such a lack of inhibition would lead to a large fraction of the population becoming active, and that slowly increasing inhibition during map formation would cause a monotonic reduction in active neurons. This has the same effect as the more artificial annealing process implemented algorithmically in abstract models of map formation.

We approximated this mechanism in our simulations by slowly increasing the GABAergic conductance of synapses onto excitatory cells, from zero to a plateau value. This plateau is reached one fourth of the way through the simulation (see Table A3). This mechanism had the desired effect. Early during map development, nearly one-quarter of the neurons in the network responded to each stimulus. This number was reduced to a small fraction of the neurons when inhibition reached its maximal level, and the active population remained small for the remainder of the training period (data not shown).

**Figure 3:**
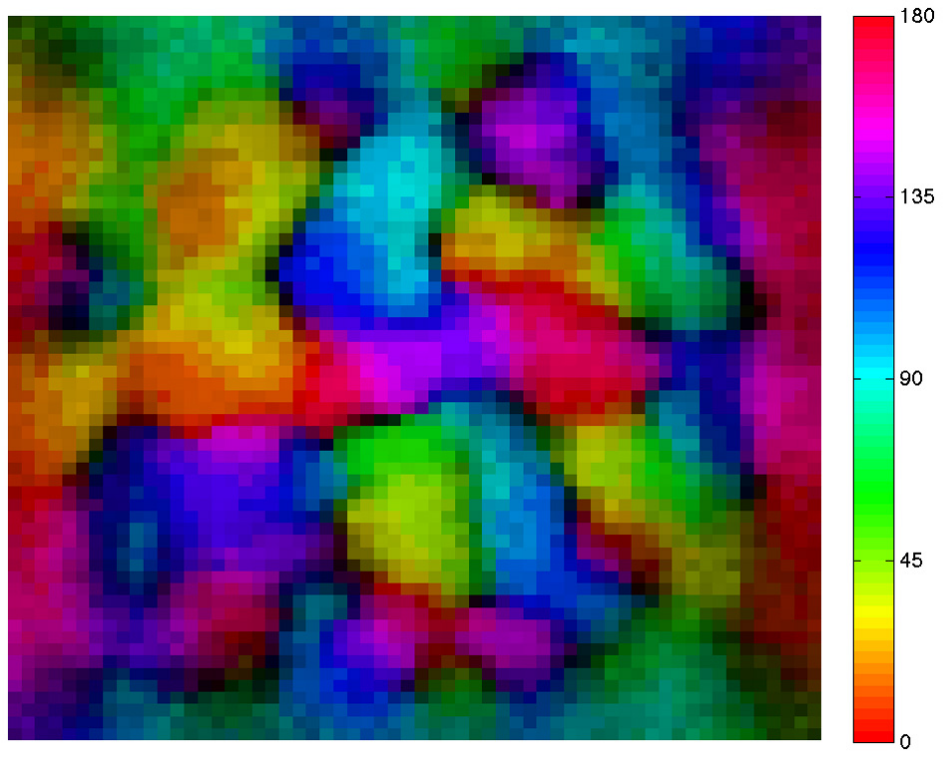
**A simulated neural network develops a smooth orientation map similar to those of cat and primate visual cortex.** The map shows the preferred orientation of individual excitatory neurons arrayed in a 60 × 60 neuron grid. Pixel colors relate location of each neuron to its preferred orientation as indicated by the color bar at right of the map. Adjacent neurons in the network tend to have similar orientation preferences. Brightness varies with orientation selectivity (dark = low selectivity, bright = high selectivity); dark areas are found at the centers of so-called orientation pinwheels.

Finally, we tested the proposed annealing mechanism in conjunction with the CAS architecture for the ability to develop smooth maps. As shown in the resulting map (Figure 3), nearby neurons in the network tend to have similar orientation preferences, i.e., the map is smooth, a characteristic of the primary visual cortex of cat, ferret, tree shrew, and monkey (Obermayer et al., 1990; Kaschube et al., 2010). In addition, dark areas are found at the centers of so-called orientation pinwheels, around which cells responding to all of the different orientations are found. The fact that each color occurs multiple times in the map reflects the fact that groups of cells respond to all orientations at each location in the visual field. This simplified spiking model based on the visual cortex develops orientation columns qualitatively similar to those found in the animal species mentioned above.

### Learning Hand-eye Coordination in a BBD controlled by a large scale spiking network

The work of Davison and Frégnac (2006) demonstrated that STDP could be used to establish a mapping between two spiking networks with correlated spiking activity. We confirm that this finding holds in a real-world task in a large-scale model of approximately 7000 spiking neurons, which was able to learn a mapping from visual targets to motor actions in a BBD.

To do so we coupled together two CAS networks to create a system that could learn the correlations between individual maps. After training, the output of a system of such networks controlled behavior in a real-world task: reaching to targets within the visual field of a BBD. To do this, we integrated a CAS-network motor map in a BBD. This motor map gave rise to autonomous arm movements, a form of “motor babbling.” With experience, this system came to correlate the location of the hand in its own visual field to the motor command needed to maintain the hand at that location, i.e., hand-eye-coordination.

**Figure 4:**
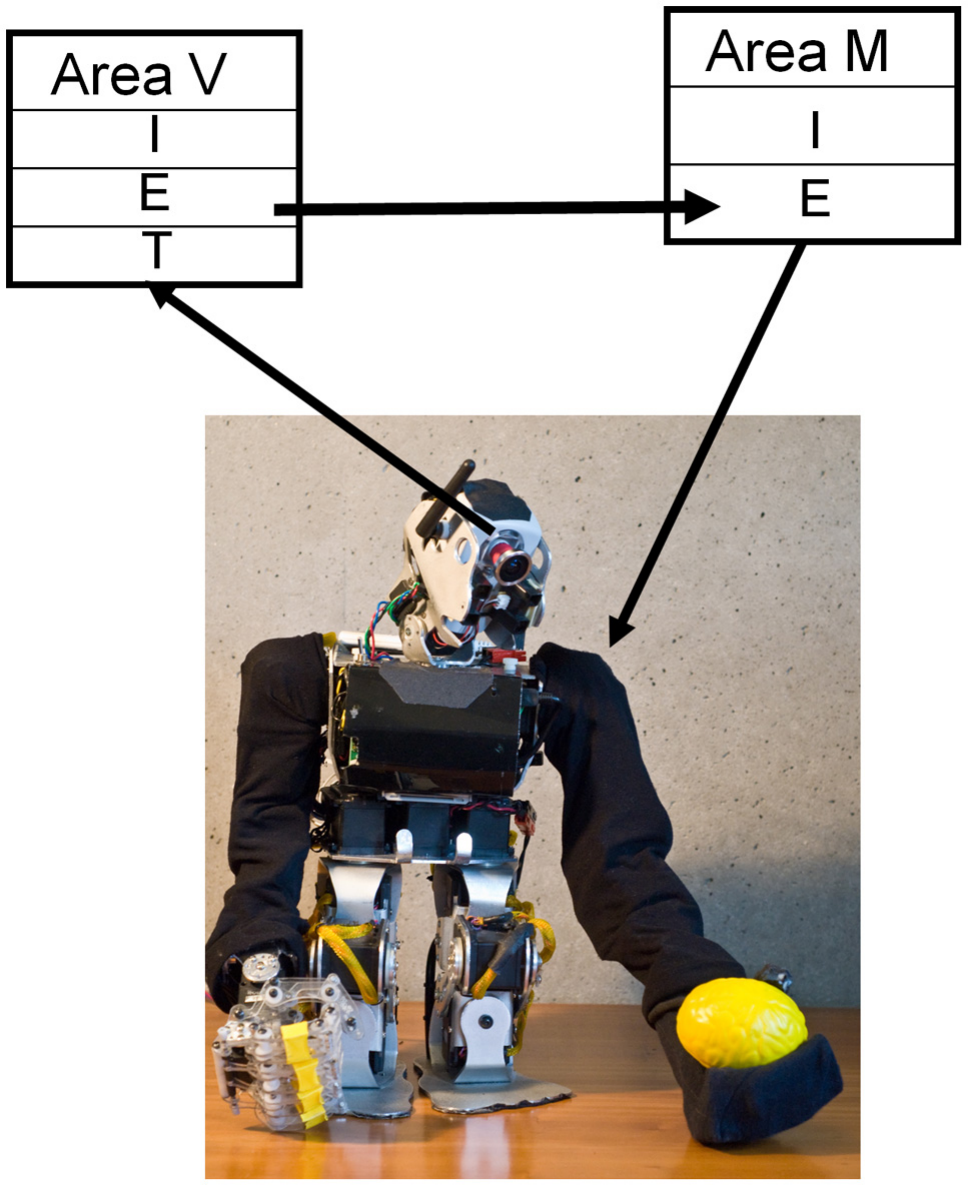
**STDP plus synaptic scaling forms a mapping between visual and motor maps.** CAS networks were used in a humanoid BBD to demonstrate that such a system could learn sensorimotor coordination. CAS networks consist of populations of excitatory (E) and inhibitory (I) neurons synaptically coupled as described in the text. Visual input from the video camera provided patterned input to “thalamic” (T) neurons of the visual area (V), while the output of excitatory neurons in the motor area (M) were used to control the two shoulder joints of the left arm. After repeatedly stimulating the motor area in one of nine different locations, and thus moving the arm to one of nine different postures, a mapping formed from the visual area responses to the location of the hand to the motor area output that drove the hand to those locations.

The upper torso of the BBD maintained a seated posture that allowed a sufficient range of arm motion (see Figure 4). The head unit containing a gray-scale video camera was aimed and held fixed during the experiment to allow the full range of motion of the left arm to fit into the camera's field of view. A bright yellow object (5 × 5 × 7 cm) attached to the end of the left arm allowed the visual system to detect the location of the end effector. The neural simulation controlled only the two shoulder joints of the BBD. Any given combination of the two joint angles yielded a unique arm posture and thus determined the location within the visual field of the bright object. The goal was to form a mapping between the visual input and joint angle commands that gave rise to that input.

The neural network controlling the behavior of the BBD consisted of the visual map area (V) and the motor area (M). Area V was a two-dimensional array of 3481 excitatory and 900 inhibitory neurons. The network formed a topographic map of the visual input from the camera (see Appendix for details of visual input processing). The activity of each neuron in this array was roughly proportional to the brightness level of the corresponding pixel from the video input.

Area M, the motor area, contained 1600 excitatory and 400 inhibitory neurons. Each excitatory neuron was assigned a preferred set of angles for each of the two shoulder joints. Nearby neurons in this predetermined map responded to similar joint angles, but different patterns of activity among these cells could evoke all possible positions of the left arm. In order to translate from neuronal firings to joint angle in the left shoulder of the BBD, the output of these cells was pooled using population vector averaging (Georgopoulos et al., 1986). That is, for each joint, the preferred joint angles of all cells, weighted by the corresponding firing rate, were summed to determine an equilibrium posture. Joint angles were recalculated in this manner, and the angles of the shoulder joints were adjusted every 250 ms.

To learn the mapping from visual input to motor output, area V was connected to area M with initially random one-way synaptic connections. In order to allow arbitrary mappings to form, the connections were all-to-all. STDP was calculated as described in “Materials and Methods” and was used to adjust the synaptic strengths during the learning process; short-term synaptic plasticity was used as described previously (Izhikevich and Edelman, 2008). In addition, the sum of the incoming synaptic strengths for each neuron was normalized to a constant value on this connection pathway. Table A4 gives all parameters used in this experiment.

In order to train the device to reach, a so-called motor-babbling reflex was incorporated in the BBD. During each movement trial of the training phase we directly stimulated one of nine different spots in the motor network by injecting current into excitatory neurons for 450 ms. This effectively drove the arm into a corresponding posture in open-loop fashion within approximately 100 ms, and the arm remained in a constant posture for nearly 400 ms. before the beginning of the next trial. A total of 15 repetitions, each generating nine postures, were used during this motor-babbling phase. During this time, STDP modulated the strength of connections between co-active neurons in the simulated visual and motor cortex, generating the visuomotor mapping.

**Figure 5:**
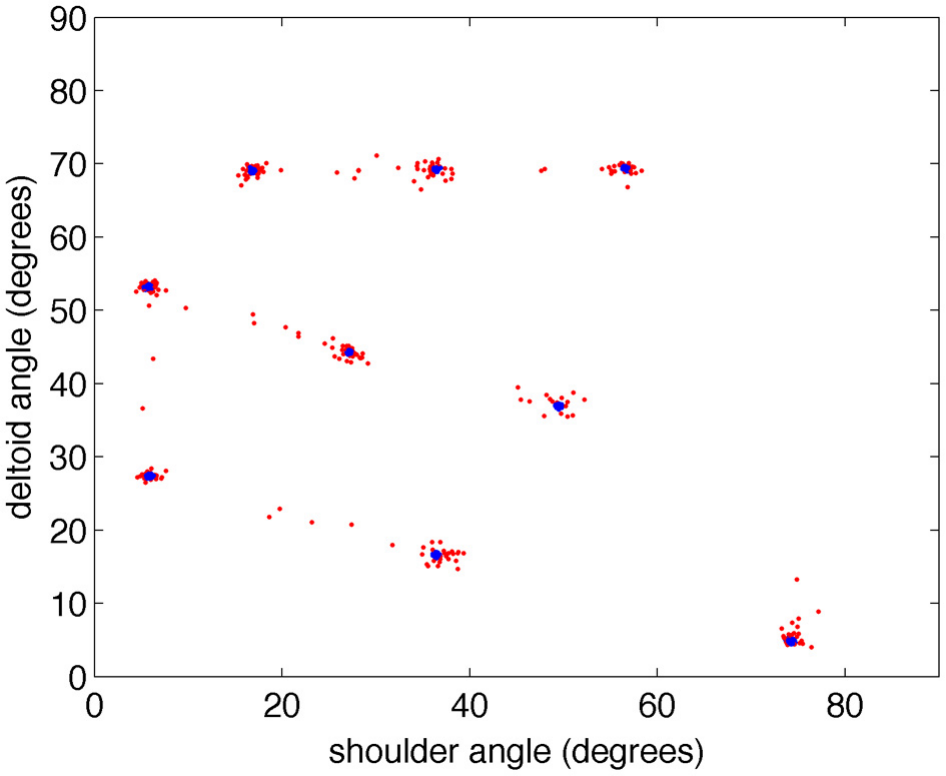
**The BBD reaches accurately toward visual targets after training.** During the testing period, the arm consistently moved in response to the visual stimulus. To demonstrate the accuracy of the movements, the joint angles of the commanded movements made during training (blue) and testing (red) are plotted in two-dimensional joint angle space every 200 ms for all five subjects. Note that the joint angles achieved during testing cluster around those achieved in the training period showing the accuracy of the visually guided, learned movements.

After the training phase, direct motor cortex stimulation was turned off, and the target yellow object was detached from the BBD. With the arm of the BBD at its side, the target object was repeatedly placed by the experimenter in each of the nine spatial locations that it had occupied during training. This experiment was repeated five times; in each repetition, parameters and conditions were unchanged, except for initial synaptic strengths and connectivity that were controlled by a seed of the random number generator function from the standard C library (Kernighan and Ritchie, 1988). During the testing period, the arm moved in response to each new visual stimulus. Figure 5 shows the joint angles that correspond to the nine successive postures assumed by the BBD during training (blue) and testing (red) phases for all five experiments. The joint angles arrived at during testing cluster around those achieved in the training period, indicating an accurate mapping between visual and motor responses. To quantify the precision of equilibrium postures, a measure of the position error was recorded. We define position error at a given joint as the difference between the joint angles of the visually evoked postures during testing and those recorded during the training period (see “Materials and Methods”). The median joint angle error, pooled across the two joints and across subjects, was 0.3°; the maximum error was 13.6°. Variability in manually positioning the stimulus in the visual field of the robot contributed to the variability in the motor error. A video clip showing the behavior of the system after being trained to reach to four positions is available in the online Supplementary Material.

## Discussion

Our studies indicate that large-scale simulations of networks of excitatory and inhibitory spiking neurons incorporating CAS anatomy and synaptic plasticity can generate dynamically stable behavior. Such networks are versatile, as shown by their ability to form smooth maps, and they can serve as a basis for systems that learn sensorimotor coordination.

How did competitive interactions in a network of spiking neurons lead to a network that can categorize external inputs? Initial synaptic strengths were randomly distributed, so neurons were not tuned to specific stimuli. For any particular pattern of input, some local population of neurons will, by chance, be slightly more responsive than alternative groups, and active neuronal groups will suppress activity in surrounding neurons. The operation of STDP then acts to increase the synaptic drive from that input pattern of activity. In addition, STDP and synaptic normalization force heterosynaptic reduction in the strength of synapses from uncorrelated input patterns.

The model networks described in this paper rely upon the presence of short-range excitation and long-range inhibition. This result is consistent with recent theoretical arguments that long-range inhibitory interactions are critical for cortical map formation (Kaschube et al., 2010). Among the three different connectivity topologies we analyzed, it was expected that the standard center-surround architecture would have also produced WTA network behavior (Dayan and Abbott, 2001). However, only the CAS inhibition motif gave rise to the generation of localized persistent activity that characterizes a WTA state. It is possible that connection architectures other than the ones we tried might produce WTA behavior. Although we did explore the parameter space for the standard center-surround model as we did for the CAS model in Figure 2A, it is also possible that even this connectivity might work under different parameter settings. It may prove informative to further explore analytically and empirically why the center-surround inhibition failed to produce WTA behavior in our simulations, and why the CAS architecture produced robust WTA behavior under these same conditions.

We have demonstrated the establishment of a mapping between two maps given spiking input from the real-world. The work of Davison and Frégnac (2006) demonstrated that STDP could be used to establish a mapping between two areas with correlated spiking activity. We confirm that this finding holds in a real-world task which, in our large-scale visuomotor model with approximately 7000 spiking neurons, was able to learn a mapping from visual targets to motor actions in a BBD. Since STDP requires consistent firing of presynaptic before postsynaptic neurons to potentiate synaptic efficacies, one might not expect that STDP would strengthen synapses from the visual to the motor area, given that motor commands occur well before any visual feedback from the arm movement occurs. However, at high firing rates STDP is purely facilitory, so that all that was required to learn the mapping between visual and motor areas was a brief overlap between the time of bursts of spikes in the two areas. This was accomplished by maintaining the BBD in each posture long enough to assure that both motor area and visual area achieved equilibrium.

In the simulated network reported here at least one type of inhibitory neuron strongly inhibits an annulus in its surround while not inhibiting nearby neurons. This differs from computational models in which inhibitory connection profiles have a Gaussian distribution with the strongest inhibition occurring within the neighboring region (Dayan and Abbott, 2001; Laing and Chow, 2001). Such models are capable of WTA behavior because strong local excitation is greater than local inhibition, essentially removing that local inhibition. In our spiking model, however, we did not obtain WTA behavior with strong local inhibition. This may relate to the previous finding that spike synchronization can prevent competition in networks of spiking neurons (Lumer, 2000). Our simulations are in agreement with this finding (see for example, Figure 2D). In addition, we have shown that WTA behavior can arise in large-scale spiking networks even in the presence of strong initial synchronization, if inhibitory neurons inhibit in an annular surrounding region rather than locally. We have found that WTA behavior still emerges in our CAS network when we shrink the inner radius to zero indicating that some level of local inhibition may be tolerated (data not shown).

Our model with annular-surround inhibition also appears to conflict with anatomical connections observed among certain inhibitory cells within the cortex. Reports of high connection probabilities between nearby basket and inhibitory neurons (Holmgren et al., 2003) and the finding that small basket cells tend to project little more than 100 μ from the cell body seem at odds with our model. However, local connections from small basket cells may perform a different role than do large basket cells that project up to 1 mm from their cell bodies, and that have been reported to mediate lateral inhibition in cortical networks (Crook et al., 1998). Regardless of the mechanisms, our simulations lead to the testable prediction that inhibition should be stronger in some annular region surrounding inhibitory neurons than it is within the local region from which it receives its excitatory inputs.

The behavior of our simulations demonstrates the versatility of networks of simulated spiking neurons endowed with CAS connectivity and activity-dependent synaptic plasticity. Further analyses of such simulations will undoubtedly prove to be a valuable tool leading to an understanding of brain function. They may also form a useful basis for more sophisticated BBDs, and for further theoretical studies of increasingly realistic brain networks.

### Conflict of interest statement

The authors declare that the research was conducted in the absence of any commercial or financial relationships that could be construed as a potential conflict of interest.

## Figures and Tables

**Table A2:**
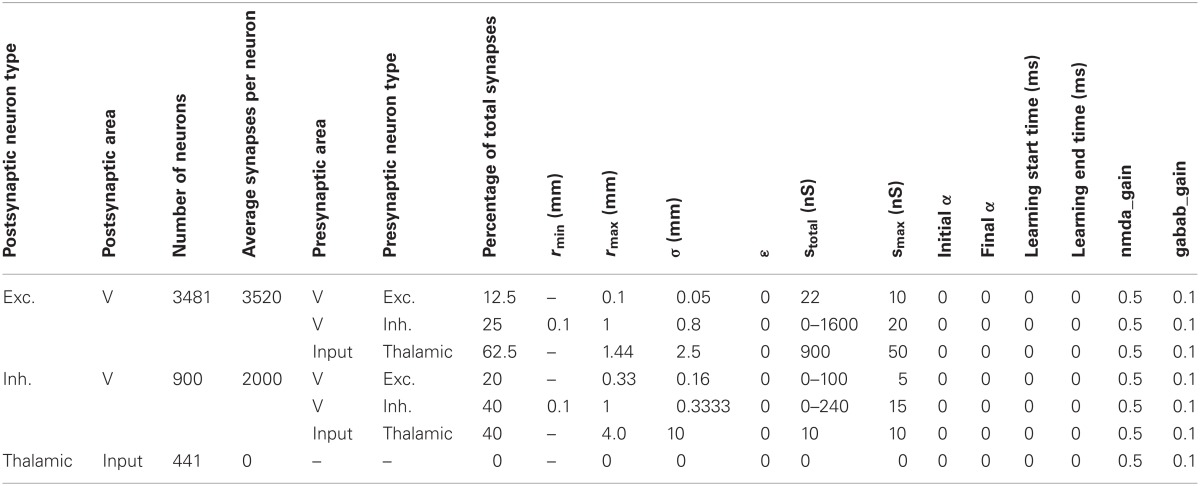
**Anatomical and synaptic parameters for the CAS network used in the parameter space analysis in results section “Spiking Activity in a WTA Network**.”

**Table A3:**
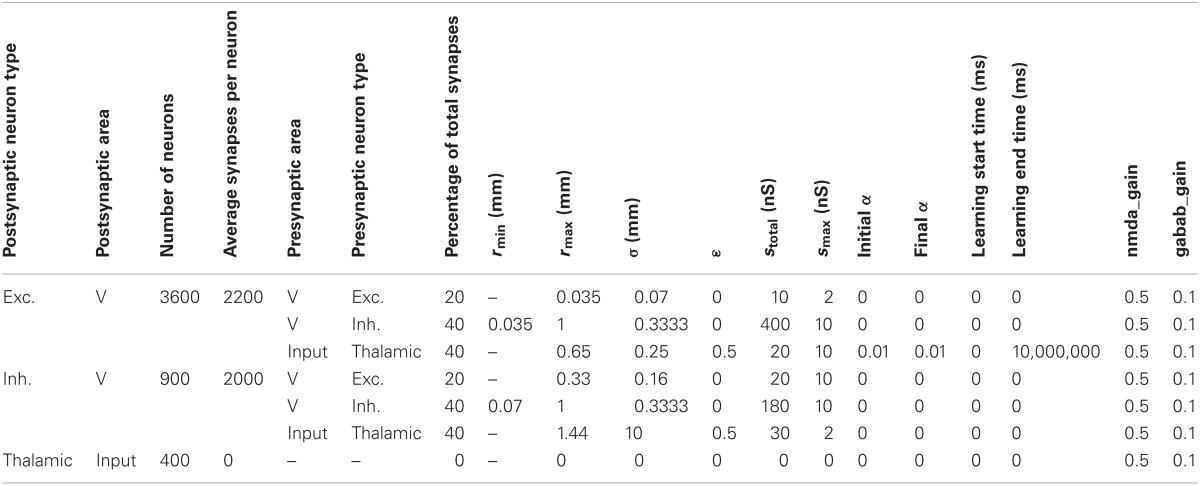
**Anatomy and synaptic parameters for the orientation selective map experiment**.

**Table A4:**
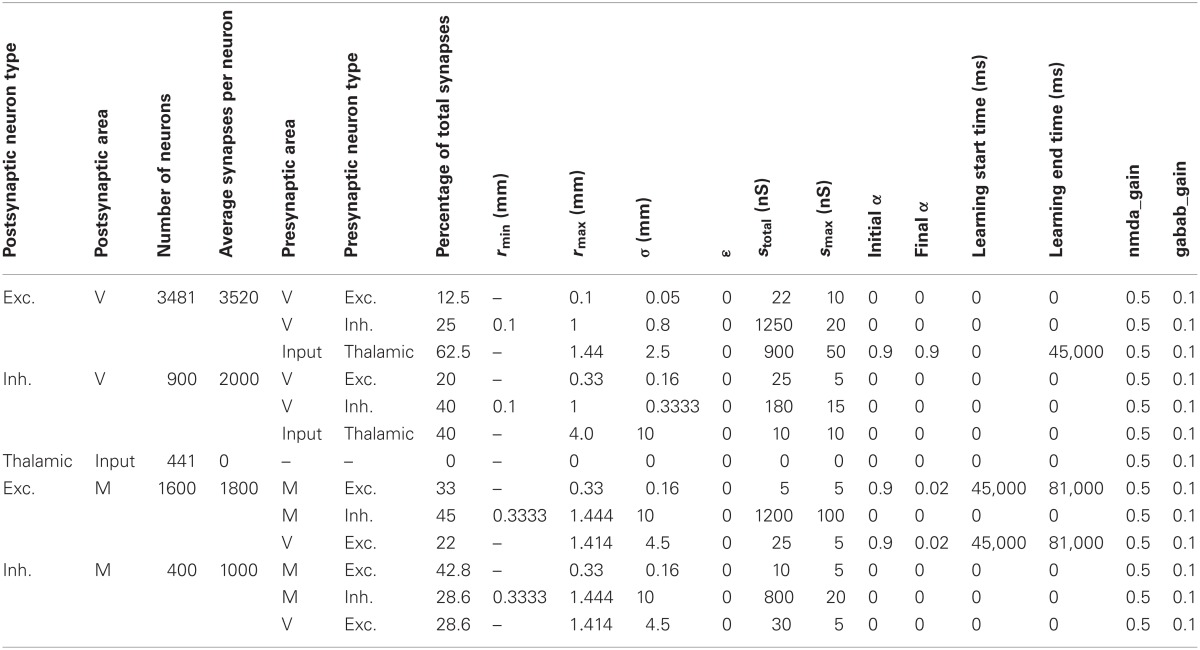
**Anatomy and synaptic parameters for the visuomotor coordination network used to control the arm of the BBD**.

## Appendix

### Neuron parameters

**Table A1:** **Neuron parameters**.

**Neuron type**	**Area**	***C***	***k***	***v*_*r*_**	***v*_*t*_**	***v*_peak_**	***a***	***b***	***c***	***d***
Excitatory	V	80	3	−60	−50	50	0.01	5	−60	10
Inhibitory	V	20	1	−55	−40	25	0.15	8	−55	200
Excitatory	Motor	100	0.7	−60	−50	0	0.03	−2	−60	100
Inhibitory	Motor	20	1	−55	−40	25	0.15	8	−55	200
Thalamic	Input	200	1.6	−60	−50	40	0.01	15	−60	10

### Anatomy

The connectivity between model neurons fell into two classes: either local-type or surround-type. For local-type connectivity, a two-dimensional Gaussian probability distribution, centered on each postsynaptic cell, determines the probability of forming a synapse between each potential presynaptic neuron within a specified maximum distance, *r*_max_

$$f(d)=ae^{-\frac{{\left(d-\mu \right)}^{2}}{2\sigma^{2}}}$$

where *a* is a scale factor set to generate, on average, a target number of synapses on each postsynaptic cell, *d* is the distance between the presynaptic neuron and the postsynaptic neuron, μ is 0, and σ is the standard deviation. In a similar manner, a two-dimensional Gaussian function was also used to specify the synaptic strength between connected neurons as a function of the distance between them in the network. The total of all synaptic efficacies was scaled to sum to a constant parameter with units in nanoSiemens (nS). Thus both connection probability and strength were maximal between nearest neighbors, and fell off as a function of distance, controlled by the same parameter, the standard deviation of a Gaussian.

For surround-type connectivity, a postsynaptic neuron receives synaptic connections from neurons located in a surrounding annular region specified by a minimum (*r*_min_) and maximum (*r*_max_) radial distance from the postsynaptic cell. (This is equivalent to saying that each presynaptic neuron sends projections to postsynaptic neurons in an annular region). The probability of forming a connection with a neuron in the annulus is determined as a function of distance from the postsynaptic cell. The function used is a Gaussian with standard deviation σ, centered at μ = (*r*_min_ + *r*_max_)/2. Thus a postsynaptic neuron connects with no neurons in the center of the annulus, has minimal connection probability at the minimum radius, increasing to the maximum probability half-way between the inner and outer radius, and falling off once again with increasing distance up to the outer radius, beyond which the connection probability is forced to zero. This probability distribution function is scaled to create a target number of synapses for each postsynaptic neuron. The synaptic strengths for the surround-type connection are also initialized using the same function, with the same parameters. However, the sum of all synaptic strengths of this type was scaled to make the total equal to a constant value under experimenter control.

In order to avoid boundary conditions in the network, the network was treated as a torus. Thus connections from neurons that would go outside of the network instead “wrap around” to connect with neurons on the opposite edge.

Table A2 shows the parameters defining the anatomy and synaptic parameters of the CAS network used in the parameter space analysis in Results section “Spiking Activity in a WTA Network.” The table defines two types of information for every neural area: the neuron composition, and the synaptic connectivity for each neuron type. The first four columns of the table list, for each separate neural population in the simulation, the type of neuron, the area in which it is located, the number of neurons in the population, and the total number of synapses per neuron.

The remaining columns define the connectivity for each type of neuron in the area. Multiple rows are necessary to define the connectivity for each postsynaptic type; one row is needed for each presynaptic neuron type forming synapses on the postsynaptic neurons. Pre-area and pre-type specify the presynaptic area and type of the neuronal group projecting to the postsynaptic group. The next column specifies the percentage of the postsynaptic cell's synapses allocated to this pathway. The remaining columns provide all of the parameters used to specify details of the synaptic pathways as described in the paragraphs above.

Table A3 shows the parameters defining the anatomy and synaptic parameters of the orientation selective map experiment in the Results section “Using CAS Architecture to Develop Maps of Orientation Selectivity”; the format is the same as that for Table A2.

Table A4 shows the parameters defining the anatomy and synaptic parameters of the visuomotor coordination network used to control the arm of The BBD in the Results section “Learning Hand-eye Coordination in a BBD Controlled by a Large Scale Spiking Network”; the format is the same as that for Table A2.

### Close up of a portion of figures 2B–D

Figures A1–A3 show close ups of Figures 2B–D.

### Control experiment parameters

The following parameters were used in control experiments to demonstrate that the CAS architecture made an improvement in WTA behavior in our simulations.

#### Standard center-surround architecture

In the classical center-surround topology, both excitatory and inhibitory neurons have local connection type but with different standard deviations. This connection architecture has been reported to produce WTA dynamics (see main text for references). In this control experiment, excitatory neurons connected with inhibitory neurons in a Gaussian distribution with maximal distance (*r*_max) of 0.33 mm and a standard deviation of 0.16 mm. Inhibitory neurons connect to excitatory neurons and themselves in a wider Gaussian distribution with maximal distance (*r*_max) at 1.44 mm and larger standard deviations (sigma = 0.8 mm) than excitatory connections.

#### Excitatory-surround, inhibitory center architecture

An additional control experiment was conducted with a connection architecture is a reverse version of our CAS topology. That is, excitatory connections are annular-surround type specified by *r*_min = 0.1 mm, *r*_max = 1 mm and a Gaussian distribution centered at (*r*_min + *r*_max)/2 with a standard deviation of 0.3333 (sigma). Inhibitory connection, on the other hand, are local Gaussian type with *r*_max = 0.333 and standard deviation of 0.16.

#### Uniform random excitatory and inhibitory architecture

In this control experiment, excitatory and inhibitory neurons have an equal probability of connecting to any other excitatory or inhibitory neuron. This is implemented as a local connection in which the maximal connection distance is set to cover the entire area (*r*_max = 1.44 mm, *r*_min = 0 mm) and standard deviation of the Gaussian distribution used to generate the connection probability is large enough to approximate a uniform distribution (sigma = 10 mm).

#### Visual input to the BBD

Video was recorded with an Axis 207MW wifi camera. Black and white images with a resolution of 320 × 240 were transmitted at 30 fps. The central portion of the video frames were used as input to a two-dimensional grid of on-center retinal ganglion cells (RGC). The grid size was 21 × 21 neurons with a center area size of 3 × 3 and the surround area of 6 × 6 neurons. Each RGC receives a current that is computed following the algorithm of Wohrer and Kornprobst (2009). These currents were constantly injected at each integration step until the next video frame was received. RGCs were modeled with the Izhikevich model (Izhikevich and Edelman, 2008) with the following parameters: *C* = 100, *Vr* = −70 mV, *Vt* = −50 mV, *k* = 1, *a* = 0.005, *b* = 0, *c* = −75 mV, *d* = 250, and *V*peak = 10 mV.

### Supplementary video

The online supplementary material includes a video showing the behavior of the BBD during testing, after it has been trained to reach to four visual locations.
